# Delta-Integration of Single Gene Shapes the Whole Metabolomic Short-Term Response to Ethanol of Recombinant *Saccharomyces cerevisiae* Strains

**DOI:** 10.3390/metabo10040140

**Published:** 2020-04-03

**Authors:** Laura Corte, Luca Roscini, Debora Casagrande Pierantoni, Roberto Maria Pellegrino, Carla Emiliani, Marina Basaglia, Lorenzo Favaro, Sergio Casella, Gianluigi Cardinali

**Affiliations:** 1 Department of Pharmaceutical Sciences, University of Perugia, 06121 Perugia, Italy; laura.corte@unipg.it (L.C.); luca.roscini@unipg.it (L.R.); deboracasagrandepierantoni@gmail.com (D.C.P.); gianluigi.cardinali@unipg.it (G.C.); 2 Department of Chemistry Biology and Biotechnology, University of Perugia, 06123 Perugia, Italy; roberto.pellegrino@unipg.it (R.M.P.); carla.emiliani@unipg.it (C.E.); 3 Department of Agronomy Food natural Resources Animals and Environment (DAFNAE), University of Padova, 35020 Legnaro, Italy; marina.basaglia@unipd.it (M.B.); sergio.casella@unipd.it (S.C.)

**Keywords:** industrial yeast engineering, bioethanol, metabolic burden, Consolidated Bioprocessing, glucoamylase, multivariate analysis, FTIR, metabolomics, liquid chromatography-mass spectrometry, ethanol stress response

## Abstract

In yeast engineering, metabolic burden is often linked to the reprogramming of resources from regular cellular activities to guarantee recombinant protein(s) production. Therefore, growth parameters can be significantly influenced. Two recombinant strains, previously developed by the multiple δ-integration of a glucoamylase in the industrial *Saccharomyces cerevisiae* 27P, did not display any detectable metabolic burden. In this study, a Fourier Transform InfraRed Spectroscopy (FTIR)-based assay was employed to investigate the effect of δ-integration on yeast strains’ tolerance to the increasing ethanol levels typical of the starch-to-ethanol industry. FTIR fingerprint, indeed, offers a holistic view of the metabolome and is a well-established method to assess the stress response of microorganisms. Cell viability and metabolomic fingerprints have been considered as parameters to detecting any physiological and/or metabolomic perturbations. Quite surprisingly, the three strains did not show any difference in cell viability but metabolomic profiles were significantly altered and different when the strains were incubated both with and without ethanol. A LC/MS untargeted workflow was applied to assess the metabolites and pathways mostly involved in these strain-specific ethanol responses, further confirming the FTIR fingerprinting of the parental and recombinant strains. These results indicated that the multiple δ-integration prompted huge metabolomic changes in response to short-term ethanol exposure, calling for deeper metabolomic and genomic insights to understand how and, to what extent, genetic engineering could affect the yeast metabolome.

## 1. Introduction

Bioethanol obtained from biomass is one of the most promising biofuels to reduce dependence on oil and decrease carbon dioxide emissions [1]. The main cost in bioethanol and other bio-products production is the substrate. Therefore, to reduce the costs, the processing of cheap materials such as energy-crops, food processing residues, agricultural and forest waste is paramount [2,3,4,5,6,7,8,9,10,11].

Besides cellulose, starchy residual biomass is the most available substrate for bioethanol considering its great availability and limited price [11,12,13]. Regardless of these advantages, starch-to-ethanol processing is very expensive because of the need for bulky amounts of commercial enzymes. Therefore, more cost-effective methods are needed and the consolidated bioprocessing (CBP) could be a breakthrough technology [14,15]. However, the CBP applied to starch requires recombinant *Saccharomyces cerevisiae* strains producing sufficient quantities of raw starch hydrolyzing enzymes to ensure complete hydrolysis at a high substrate loading. This has become the primary focus of several research groups and great progress towards proof of concept in industrial strains has been made [14,15,16,17,18]. Nevertheless, engineering industrial strains for the production of amylases at titers suitable in large scale applications still remains a major challenge [14,18]. The expression of heterologous genes can induce a stressful condition, known as metabolic burden, which can affect the metabolic performances of the recombinant strain [19,20]. In the development of engineered yeast strains, metabolic burden is often linked to the synthesis of recombinant proteins, since both additional energetic costs and competition for limited transcriptional and translational resources could occur in engineered cells. As a result, growth parameters, such as biomass, yield and specific substrate consumption rate, could be significantly impaired. 

In a recent paper, two recombinant strains, *S. cerevisiae* F2 and F6, engineered for the multiple δ-integration of the glucoamylase *sGAI* from *Aspergillus awamori,* did not exhibit any detectable metabolic burden once compared in terms of ethanol production and yield to their parental 27P [21]. Based on this evidence, in this work, we investigated the effect of δ-integration on the yeast strain’s ability to withstand the increasing ethanol stressing levels typical of the starch-to-ethanol industry. Ethanol stress is indeed one of the main factors affecting the complete fermentation and higher glucose to ethanol yield in the bioethanol industry [18,22]. In order to ensure the overall ethanol yield of the process, any recombinant strains tailored for starch (or cellulose)-to-ethanol production must retain several industrial traits (i.e., ethanol tolerance, ethanol yield, biomass yield) of their parental strain. Therefore, in this paper, it seemed noteworthy to screen increasing concentrations of ethanol levels to assess the ethanol tolerance of the two recombinant strains F2 and F6 and their metabolomic reactions to short-term ethanol response. The yeast cell reacts to ethanol by quickly reprogramming many cellular activities to promote survival during the stress condition and protect important cell components, which stimulate the normal activity of the cell. The ethanol stress responses are multifactorial and comprise various features such as signalling, gene level control and accumulation of the needed protectants [23]. Although profuse efforts have been spent at genetic, transcriptional and metabolomic levels, the precise mechanism of ethanol tolerance is not completely revealed [23,24] and a more holistic and in-depth understanding of ethanol tolerance in *S. cerevisiae* is crucial to support novel yeast metabolic engineering methodologies and improve ethanol production. Metabolomics has been gradually applied in investigating the cellular stress responses to ethanol [25,26,27]. As such, metabolites in pathways of interest can be unbiasedly characterized to enlighten the effects of perturbation by metabolic profiling, which has been considered to provide useful information for yeast cells’ stress responses. 

In the present study, a Fourier Transform InfraRed Spectroscopy (FTIR)-based assay was employed to investigate the response to ethanol stress of both a parental and two recombinant *S. cerevisiae* strains. Cell viability and metabolomic fingerprints have been considered as parameters to assess any physiological and/or metabolomic perturbations in response to the increasing concentrations of ethanol. Quite unexpectedly, the three strains did not show any difference in cell viability but exhibited significantly different metabolomic fingerprints. This result indicated that the multiple δ-integration prompted huge metabolomic changes in response to short-term ethanol exposure, calling for deeper metabolomic insights to understand how, and to what extent, genetic engineering could affect the yeast metabolomic reaction to ethanol stress. A more specific metabolomic approach, based on LC/MS analysis, was then applied to investigate the nature of the differences detected by FTIR assay at metabolites level. Significantly perturbed metabolic pathways, identified by metabolomic pathway analysis, further supported the hypothesis that diverse δ-integrations, although not resulting in higher sensitivity to ethanol, completely rearranged the metabolome of the recombinant strains by inducing different over- or down-regulations of metabolite production.

## 2. Results and Discussion

### 2.1. FTIR Fingerprints Under Ethanol Stress

**Figure 1:**
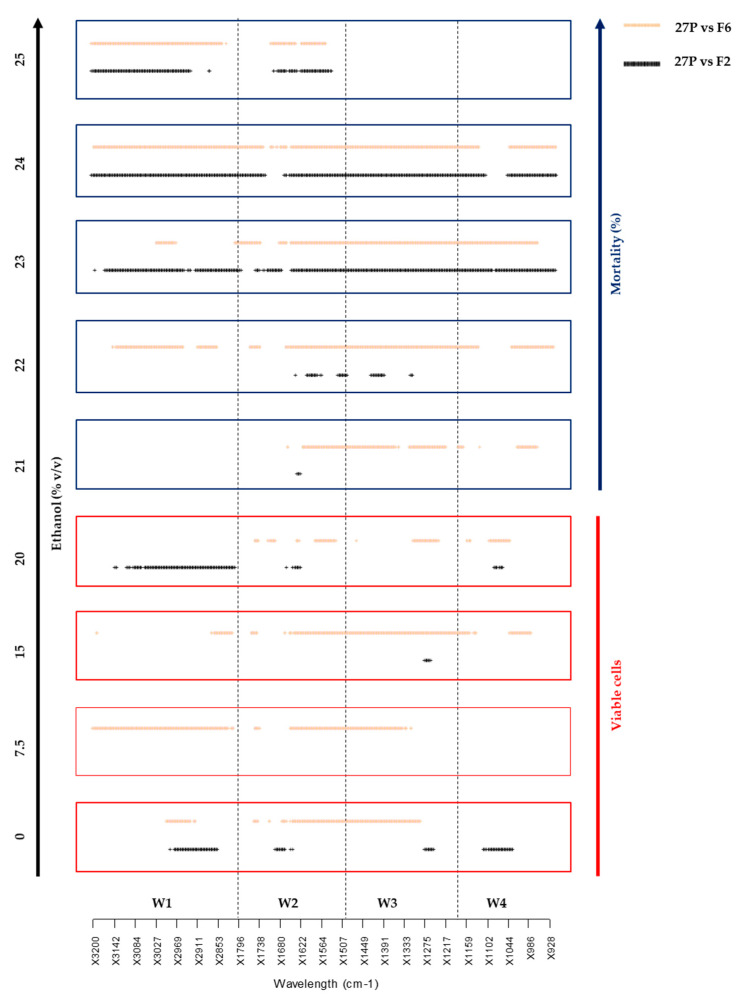
FTIR fingerprints of the parental *S. cerevisiae* 27P *vs*. recombinant strains (F2 and F6) at increasing ethanol concentrations (0%, 7.5%, 15%, 20%, 21%, 22%, 23%, 24%, 25 % v/v). **Legend.** Specific spectral areas involved in the ethanol response were considered, namely: fatty acids (W1), amides (W2), mixed region (W3) and carbohydrates (W4).

The two recombinant strains F2 and F6 were engineered to ferment raw starch into ethanol through the multiple delta-integration of the *sGAI* gene of *A. awamori* under the constitutive transcriptional control of PGK1 [21]. The evidence that both the parental *S. cerevisiae* 27P and recombinant strains displayed similar ethanol yield and growth rate from glucose indicated that the multiple delta-integration and expression of the *sGAI* gene did not result in any evident metabolic burden when strains were grown in glucose under oxygen limiting conditions [21]. In this study, a more detailed analysis has been carried out to investigate the short-term ethanol response of both parental and engineered strains. A FTIR-based assay, already employed as a powerful technique for ecotoxicological assessments [28,29,30], was carried out to estimate the type and extent of perturbations induced by ethanol stress on both parental and recombinant yeast strains. Ethanol levels were specifically chosen based on the alcohol concentrations typical of the bioethanol industry [18,22]. The FTIR spectra of yeast cells were recorded at increasing ethanol concentrations (0%, 7.5%, 15%, 20%, 21%, 22%, 23%, 24%, 25% v/v), parallel with mortality data (Table 1). Significant Wavelengths Analysis (SWA) detected all relevant differences between spectra from different experimental conditions, producing a plot in which all the statistically significant difference wavelengths (*p* < 0.01) are reported as dots (Figure 1).

In terms of cell mortality, delta-integration did not affect the response of both recombinant strains *vs*. that of the parental. The exposure to increasing concentrations of ethanol did not induce mortality up to 20% alcohol while, at higher concentrations, cell mortality increased proportionally with the increase in concentration (Table 1), suggesting that the detoxification mechanisms were active up to 20% ethanol, whereas at higher dosages the resulting mortality was concentration-dependent. 

*Viceversa*, the comparison between FTIR fingerprints revealed that δ-integration produced an unbalance in the metabolome of recombinant strains already in resting conditions (0% ethanol), as depicted by the significantly different patterns of Figure 1. In general, the response of the recombinant F6 was always different from that of the parental (orange dots), regardless of cell mortality (Table 1). Conversely, the FTIR fingerprint of *S. cerevisiae* F2 did not diverge from that of the parental for up to 20% ethanol, showing a different response only from 23% ethanol, a concentration inducing almost 50% mortality. As such, the metabolomic fingerprinting approach gives a holistic view of the metabolome and is well-established as a method to qualify metabolomic changes in microbial cells [31,32,33] and to characterize the stress response of microorganisms [34,35]. Furthermore, it is suitable for complex experimental designs to explore multiple different conditions, allowing the selection of the most significant ones for more in-depth metabolomic or other -omic approaches.

### 2.2. Metabolomic Changes Induced by δ-Integration 

The differences detected by FTIR were deeper investigated through LC/MS analysis. LC/MS was carried out only on cell extracts from controls (0% ethanol) and from 20% to 24% ethanol (v/v)-treated cells, triggering increasing mortality values of up to nearly 80% (Table 1). Metabolomic analysis based on LC/MS identified a total of 143 metabolites with known structures (Supplementary Table S1). This is the first report describing the metabolites produced by resting *S. cerevisiae* cells as a short-term ethanol response, whereas the metabolomic insights reported to date on ethanol stress in *S. cerevisiae* were applied only on cells growing in the presence of ethanol [23,25,26,27].

**Figure 2:**
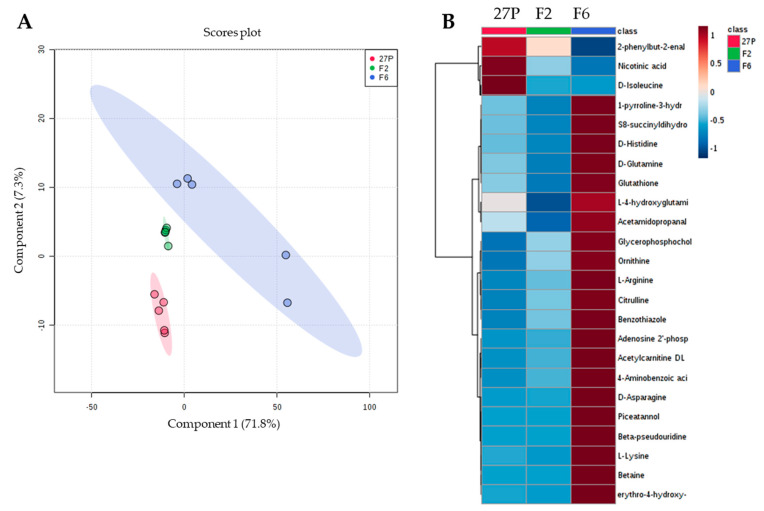
PLS-DA scores plot showing differences between the not ethanol-treated cells of the parental *S. cerevisiae* 27P and the recombinant F2 and F6 strains (**A**) and resulting heat map of the top 25 significantly changed metabolites selected by VIP scores (**B**). **Legend.** PLS-DA scores plot of PC1 vs. PC2: the explained variances are shown in brackets (A). The colored boxes indicate the relative concentrations of the corresponding metabolite in each group under study. The color scale is log2 transformed value and indicates relatively high (red) and low (blue) metabolite levels (B).

Partial Least Squares-Discriminant Analysis (PLS-DA) was applied on control samples to explore the metabolic shift induced by δ-integration in recombinant yeasts (Figure 2A), as suggested by the FTIR patterns of the not-treated cells of Figure 1. PLS-DA [36] is a PLS regression method [37] which correlates a series of response variables, y to a series of predictor variables, X, where y is a set of binary variables of describing the categories of X [38]. PLS-DA is highly efficient in the estimation of the independent original X-values (called latent variables, LV), which correlate optimally with the detected changes in the dependent variable, y [38]. The tight clustering of replicates confirmed the high reproducibility of the method. *S. cerevisiae* 27P, F2 and F6 were clearly distinguishable in the score plot generating by combining PC1 (71.8% of the total variance) and PC2 (7.3% of total variance), confirming that the *sGAI* δ-integration induced an alteration in the metabolomes in both recombinants compared to that of the parental strain. To explore the more influent variables in the PLS-DA model, Variable Importance in Projection (VIP) scores were calculated. VIP scores are a weighted sum of squares of the PLS weights for each variable and assess the input of each predictor variable to the model [37]. It is frequently used as a parameter for variable selection [38,39]. Since the average of squared VIP score is equal to 1, the ‘greater than one’ rule is used as a criterion for variable selection [39]. According to the VIP scores from PLS-DA analysis (Supplementary Table S2), Hierarchical Clustering Analysis (HCA) was performed to highlight the variation trend of differential metabolites between strains (Figure 2B).

Differential altered metabolites clustered separately. Specifically, three metabolites in the upper cluster (2-phenylbutil-2enal, nicotinic acid and D-isoleucine) were up-regulated in the parental strain and down-regulated in both recombinants. Conversely, the other 21 metabolites in the lower cluster, including mainly amino acids, were more overexpressed in F6 samples than in the other two strains. The multiple δ-integration of the glucoamylase *sGAI* induced a differential shaping of cell metabolites in the two recombinant strains, exerting a stronger effect in *S. cerevisiae* F6. One possibility to explain these differential alterations in cell metabolomes in resting conditions is to consider that the δ-integration occurred differently in the two strains. This hypothesis is supported by the different starch-hydrolyzing abilities of the two recombinants, with *S. cerevisiae* F2 displaying higher values [21]. Although the δ-integration did not induce any metabolic burden in the two recombinants [21], the significant alterations detected in the metabolome of *S. cerevisiae* F6 would suggest that it can directly affect some parental genes and/or promoter sequences in way that has yet to be understood. Conversely, the higher similarity between parental and recombinant F2 metabolomes could be attributable to an alteration induced indirectly and/or in part compensated by the reshuffling of the cell metabolome. Deeper genomic and transcriptomic insights are in progress to elucidate these fascinating hypotheses. 

### 2.3. Changes in Intracellular Metabolites in the Short-Term Response to Ethanol of the Recombinant F2 and F6 S. Cerevisiae Strains 

**Figure 3:**
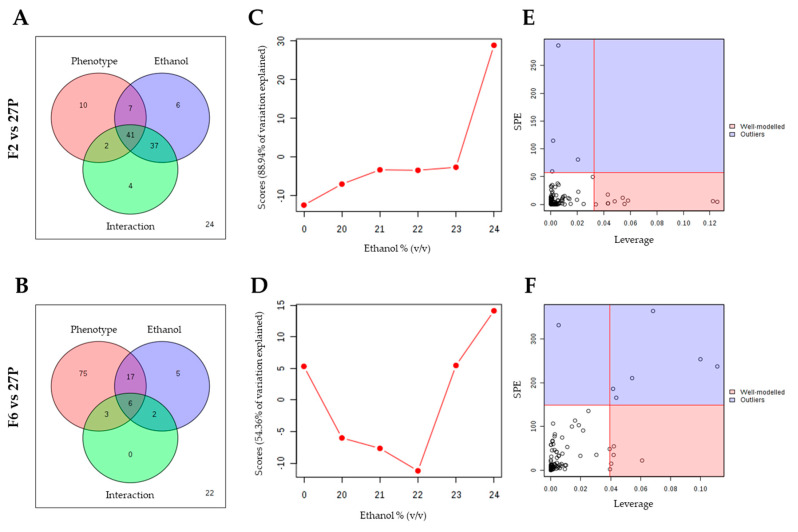
Two-way ANOVA (**A**, **B**) and ANOVA-Simultaneous Component Analysis (ASCA) (**C**-**F**) on metabolites produced by yeast cells (F2 *vs*. 27P and F6 *vs*. 27P) exposed to increasing ethanol concentrations (0%, 20%, 21%, 22%, 23%, 24 %, v/v). **Legend.** Venn diagram of metabolites affected by ethanol concentration, strain and their interaction (A,B). Major pattern of metabolites associated with ethanol concentration (C,D). ANOVA-Simultaneous Component Analysis (ASCA) selection of important metabolites associated with ethanol concentration by Leverage/SPE analysis (E,F). Ten and four metabolites were identified as responsible for the observed variation in the comparison between parental and recombinants F2 (E) and F6 (F), respectively.

The short-term responses of δ-integrated strains to increasing ethanol concentrations was investigated by comparing the parental strain with each recombinant, separately. Two-way ANOVA was used for comparative analysis between different groups to find the differential metabolites based on *p* value (*p* < 0.05). In the first comparison, between the parental and the recombinant F2 (Figure 3A, Supplementary Table S3), the number of differentially altered metabolites significantly affected by *i.* phenotype, *ii.* ethanol concentration and *iii.* their interaction, which was 60, 91 and 84 respectively, with 41 metabolites simultaneously influenced by all these factors. Conversely, in the comparison between the parental and the recombinant F6, 101, 30 and 11 differentially altered metabolites were affected respectively by *i.* phenotype, *ii.* ethanol concentration and *iii.* their interaction, respectively, with six metabolites simultaneously influenced by all these factors (Figure 3B, Supplementary Table S4). 

The major patterns associated with each factor have been identified through ANOVA-Simultaneous Component Analysis (ASCA). ASCA revealed that, in both cases, 100% of the observed variations could be explained by the main effect of the phenotype (Supplementary Figure S1), while the 88.9% and 54.36% variance in F2 and F6 could be explained, respectively, by the main effect of ethanol exposure (Figure 3C,D). Ethanol concentration score plots based on PC1 of the corresponding sub-models showed that the scores gradually increased along the concentration gradient considered (Figure 3C). This indicates that ethanol induced a dose-dependent activation of the metabolomic activity of the F2 recombinant with respect to that of the parental. A different trend was observed for metabolomic changes between parental and recombinant F6 (Figure 3D): the scores firstly gradually decrease up to 22% v/v ethanol and then rapidly increase at higher ethanol levels, indicating that opposite alterations occur as ethanol concentration increases. The large variance contribution of the interaction effect confirms that metabolic changes induced by the exposition at increasing ethanol levels were different between the parental and each recombinant strain (91.76% and 47.08% variance) (Supplementary Figure S1). 

Finally, the major trends associated with each experimental factor and their interactions has been identified through Leverage/SPE analysis. Variables with a low SPE and higher leverage had a significant contribution to the model and were picked out as influentially affected compounds (Supplementary Tables S5 and S6). Specifically, the number of differentially altered metabolites responsible for the observed variation in response to ethanol were ten (Adenosine, D-Isoleucine, Acetamidopropanal, L-Phenylalanine, Betaine, D-Glutamine, Beta-pseudouridine, Piceatannol, L-Arginine, D-Methionine) in the comparison with *S. cerevisiae* F2 (Figure 3E, Supplementary Table S5) and four (L-4-hydroxyglutamic semialdehyde, Nicotinic acid, L-Lysine, Acetamidopropanal) in the comparison with the F6 strain (Figure 3F, Supplementary Table S6). Most of these metabolites were aminoacids or intermediates in aminoacids’ metabolism, such as L-4-Hydroxyglutamate semialdehyde and acetamidopropanal, suggesting that δ-integration impacted the aminoacid metabolism of recombinant strains when exposed to ethanol. This different aminoacids’ level may have occurred as a result of increased protein degradation [40], already reported as a general stress response under oxidative stress, or following the membrane function disruption affecting the delivery of aminoacids into the cell [41].

### 2.4. Metabolic Pathway Involved in the Response to Ethanol Stress of Engineered Strains F2 and F6.

**Figure 4:**
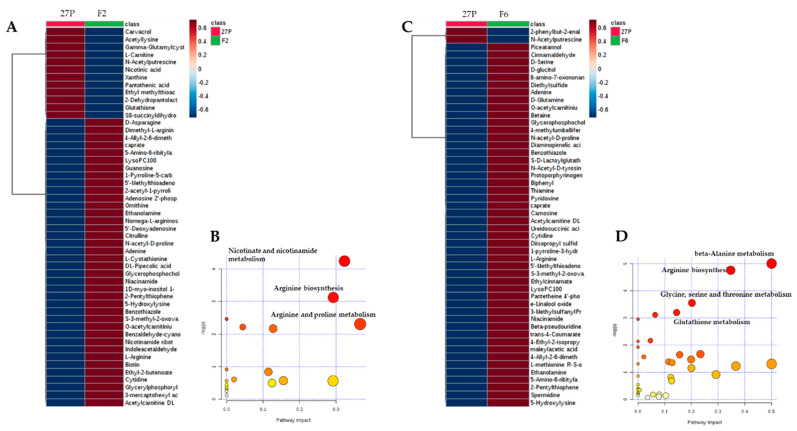
Heatmap of the top 50 metabolites significantly changed after exposure to increasing concentrations of ethanol (20–24% v/v) between the cells of 27P compared to those of F2 (**A**) or F6 (**C**). The analysis of the most important metabolomic pathways differentially perturbed by ethanol stress is also reported for F2 (**B**) and F6 (**D**) cells. **Legend.** y-axis: -log(p) represents metabolic pathways containing metabolites that are significantly different between parental and the recombinant strain. x-axis: pathway impact represents the impact of these significantly changed metabolites on the overall pathway, based on their position/number within the pathway.

HCA was employed to highlight the variation trend of differential metabolites in the comparisons between 27P and each recombinant at increasing concentrations of ethanol (20–24% v/v). The metabolites for HCA were selected based on *t-*test results (*p* < 0.05) (Supplementary Table S7,8). Both recombinants exhibited a strain-specific pattern in response to ethanol stress (Figure 4A,C) with metabolites clustered into two distinct groups. Most of them, including the aminoacids (i.e., lysine and arginine) or metabolites involved in the biosynthesis of aminoacids or glycerophospholipids, were up-regulated with respect to the parental strain, suggesting that δ-integration induced some different metabolic circuits in response to ethanol stress. Conversely, 12 metabolites in the recombinant F2 and 2 metabolites in the recombinant F6 resulted down-regulated compared to the parental, with N-acetylputrescine, involved in the arginine and proline metabolism, common to both responses.

Global test and relative betweenness centrality algorithms were then conducted for pathway enrichment analysis and pathway topology analysis, respectively. According to the results of significance of the perturbed metabolic pathway and the centrality of metabolites in the metabolic pathway, the perturbation of several metabolic pathways was found to be significant. Specifically, arginine and proline metabolism, nicotinate and nicotinamide metabolism and arginine biosynthesis resulted in alterations in the F2 recombinant, with an impact of 0.37, 0.32 and 0.29, respectively (Figure 4B, Supplementary Table S9). On the contrary, the main impacted pathways in the F6 recombinant were beta-alanine metabolism, arginine biosynthesis and glycine, serine and threonine metabolism and glutathione metabolism, with an impact of 0.50, 0.35, 0.20 and 0.14, respectively (Figure 4D, Supplementary Table S10).

Both recombinant strains up-regulated arginine, citrulline and ornithine (Figure 4A,C), thus impacting on the pathway of arginine biosynthesis. This is in agreement with previous studies reporting that, in *S. cerevisiae*, arginine [42] and other aminoacids (i.e., glutamate, proline and tryptophan) play a protective role against ethanol stress by maintaining the integrity of the cell wall and plasma membrane [36,42]. Moreover, the alteration in the arginine biosynthesis can trigger the TCA cycle, thus providing more energy to contrast ethanol stress [43]. The engineered strain F2 also altered nicotinate and nicotinamide and arginine and proline metabolisms, deeply involved as antioxidants in the cell redox status of *S. cerevisiae* [42,44,45,46]. This observation would imply that cells need to activate the arginine biosynthesis pathway to reduce stress. 

In the case of *S. cerevisiae* F6, the effect of δ-integration produced a completely different perturbation in the metabolic pathways (Figure 4D). The disturbance of the beta-alanine metabolism was found to be the most important, with a significant increase in spermidine, beta-alanine and L-aspartate (Supplementary Figure S2). Beta-alanine metabolism is a metabolic route involving several compounds bound to oxidative stress reaction, such as spermidine, spermine or aminopropanal. Many studies demonstrated the positive influence of spermidine and spermine on yeast fermentation and growth ability in the presence of ethanol [44,47]. The modulation of spermine content is also linked to enhanced tolerance to lignocellulose-derived inhibitors [48]. All other pathways detected for F6 recombinant are directly involved in glutathione metabolism. Glutathione is one of the most essential redox-scavenging compounds in yeast cells. The ratio of the two different forms, reduced (GSH) and oxidized (GSSG, glutathione disulfide) and the GSSG:GSH rate predicts the physiological and oxidative state of the cell, with healthy cells having lower GSSG:GSH values [49,50,51]. The biosynthesis of great amounts of glutathione correlates with the yeast oxidative stress resistance. In fact, glutathione participates in several functions in cells, especially searching for free radicals (ROS) present in the cytosol [52]. In addition, a higher viability is often linked to high glutathione levels in yeast cells [53]. Moreover, glutathione controls membrane dynamics and counteracts ROS effects linked to the fluidized membrane, such as peroxidation and de-esterification of its lipidic fraction [54,55]. Together with the thioredoxin system, glutathione is also involved in an inducible adaptive response mechanism, able to permit oxidative stress resistance [52]. 

Finally, all pathways induced by δ-integration in the two recombinants are interconnected through the NAD/NADH metabolism: aminoacids biosynthesis or accumulation in response to stress as well as glutathione oxidation/reduction are all mediated by continuous switching between the oxidized and reduced NAD form [34,44,56].

## 3. Materials and Methods 

### 3.1. Cultures and Growth Conditions

The yeast strains used in this work are: *S. cerevisiae* 27P, an industrial yeast, and two recombinant strains, namely F2 and F6, developed through the delta-integration of the *sGAI* gene of *A. awamori* into the 27P chromosomes [21]. Cultures were inoculated at an optical density at 600 nm (OD_600_) of 0.2 in 500 mL bottles with 50 mL of fresh YPD medium (yeast extract 1%, peptone 1% and dextrose 2% Difco Laboratories, USA) and grown at 25 °C under shaking at 150 rpm. Cell growth was monitored by determining OD_600_ and stopped after 18 h. Each culture was prepared for an FTIR-based bioassay, as detailed in the following paragraph.

### 3.2. FTIR-Based Bioassay

An FTIR-based assay for stress response analysis was carried out according to the procedure proposed by Corte and colleagues [34]. Each suspension was centrifuged (5 min at 5300× g), washed twice with distilled sterile water and re-suspended in High Performance Liquid Chromatography (HPLC) grade water to obtain an optical density of OD_600_ = 50. Each cell suspension was distributed in 15 mL polypropylene tubes, one for each tested concentration of the chemicals. In each tube, 5 mL cell suspension and 5 mL double-concentrate ethanol solution were pipetted to obtain the final concentrations of 7.5%, 15%, 20%, 21%, 22%, 23%, 24% and 25% (v/v) and a uniform cell density at OD_600_ = 25. Control (0% ethanol concentration) was obtained by re-suspending cells in distilled sterile water. All tests were carried out in triplicate. Polypropylene tubes were incubated for 1h at 25°C in a shaking incubator set at 50 rpm. After the incubation, 1.5 mL of each cell suspension were centrifuged (5 min at 5300× g), washed three times with distilled sterile water and resuspended in HPLC grade water to reach the final concentration of 2.5 × 10^8^ cells mL^−1^. For each condition, 105 μL volume was sampled for three independent FTIR readings (35 μL each, according to the technique suggested by Essendoubi and colleagues [57]) while 100 μL were serially diluted for viability assessment. Viable cell count was carried out on YPDA supplemented with chloramphenicol (0.5 g L^−1^) plates. Cell mortality (M) was calculated as M = (1 - Cv/Ct) × 100, where Cv is the number of viable cells in the tested sample and Ct the number of viable cells in the control suspension.

### 3.3. Spectra Pre-Processing

FTIR measurements were performed in transmission mode. All spectra were recorded in the range between 4000 and 400 cm^−1^. Spectral resolution was set at 4 cm^−1^, sampling 256 scans per sample to obtain high quality spectra (signal to noise ratio values greater than 4000 within the 2100–1900 cm^−1^ interval). The software OPUS version 6.5 (BRUKER Optics GmbH, Ettlingen, Germany) was used to assess the quality test, subtract the interference of atmospheric CO_2_ and water vapour, correct baseline (rubberband method with 64 points) and to apply vector normalization to the whole spectra.

### 3.4. Untargeted Metabolomics Profile Determination of Cells under Ethanol Stress by LC/MS Analysis

The metabolomic analysis of 27P, F2 and F6 samples challenged with seven increasing ethanol concentrations was performed using a LC/MS untargeted workflow. Each thesis has been tested in five replicates (n = 5), with a total of 105 samples processed. Cell suspensions, calibrated at 10^8^ cells, was centrifuged (5 min at 5300× g) and the resulting pellet was mixed with glass beads and lysed using FastPrep®-24 Tissue and Cell Homogenizer (MP Biomedicals, Irvine, CA, USA), at a speed setting of 6.0 for 120 s. The degree of cell breakage was checked microscopically. A total of 1 mL of methanol was added to each lysate, vortexed, and centrifuged at 3000 rpm for 5 minutes. Supernatants were transferred to the HPLC vials and 0.5μL was injected into the LC/MS system. LC/MS analyses were performed using an Agilent 1260 Infinity UHPLC system coupled to an Agilent 6530 Q-TOF with Agilent JetStream source (Agilent Technologies, USA). The LC consists of a quaternary pump and an autosampler with a thermostated column compartment. The whole LC/MS system was governed by Agilent MassHunter software (version B.09.00). The Ion Pairing Chromatography (IPC) method was used to achieve a wide separation of polar metabolite classes with ACME^TM^ Amide C18 column (150 × 2.1 mm, 3 μm, Phase Analytical Technology LLC, State College, PA, USA) thermostated at 50 °C. The separation of metabolites was achieved using a flow of 0.35mL min-1 of a binary gradient of 0.2% Heptafluorobutyric acid (HFBA) in water (solvent A) and 0.1% Formic Acid in Methanol (solvent B). The initial condition was 2% of B for 2 min, followed by a transition to gradient from 2% to 80% of B in 5 min and an isocratic step of 8 min. After that, the run was stopped and the column was reconditioned for 4 min at initial conditions. An autosampler injected each sample using a needle wash program of 10 sec with methanol. Each run cycle was completed in 20 min. The ion source operated in positive ion mode using nitrogen as drying gas at 35 psi and 250 °C. The capillary was set at 2000 V with fragmentor, skimmer and octopole Radio Frequency (RF) set at 110, 65 and 750, respectively. Dynamic mass axis calibration with accuracy < 5 ppm was achieved by continuous infusion in the source of a reference mass solution (Agilent G1969-85001). The spectrometer acquired data in full-scan mode in the 50 - 1700 mass range at 1.5 spectra/sec. LC/MS raw files were aligned and processed using Batch Recursive Feature Extraction algorithm of MassHunter Profinder (Agilent B.08.00). The data of features with score > 90% were imported in Mass Profiler Software (Agilent B.08.01) to perform features annotation using the Search Database algorithm. For this purpose, the Yeast Metabolome Database [58] was adapted to work in Agilent Mass Profiler. Only annotated metabolites with a quality identification score > 90% were retained. 

### 3.5. Data Analysis

#### 3.5.1. Significant Wavelengths Analysis (SWA) Throughout the Spectra 

An R script (www.cran.org) was employed to select and display the FTIR spectral regions with statistically significant differences in the comparison between spectra of parental and recombinant strains from the different experimental conditions tested. Briefly, pairs of spectra, each with three replicas, were compared using the Student *t*-test for each wavelength separately. For each wave number, the calculated *p*-value was recorded. This operation produced, for each pair of spectra, a vector of *p*-values that were subsequently transformed in 1 (for *p* < 0.01) and 0 (for *p* > 0.01). Vectors were collected in two types of matrices, one containing the vectors for all possible pairwise comparisons, i.e., the (n^2^ − n)/2 comparisons among the n conditions under test, and the other, containing only the (n − 1) comparisons of the n conditions with the control condition. The matrix data were plotted with wave numbers in the x-axis and comparisons in the y-axis. These plots reported the presence of a wavelength with statistically significant difference (*p* < 0.01) as a dot. These operations were reiteratively carried out for each experimental condition tested.

#### 3.5.2. Metabolite Data Analysis

The sample weight and non-normalized peak areas are available in Supplementary Table S1.

Metabolomic data analysis was performed with MetaboAnalyst 4.0 [59]. Data were filtered based on Interquartile Range, and normalized to sample median and scaled by Pareto scaling. PLS-DA was applied on control samples to explore the metabolomic changes induced by δ-integration. PLS-DA models were validated by 100 permutations. Two-way ANOVA and ASCA was used to investigate the short-term responses of δ-integrated strains to short-term ethanol stress. The two-way ANOVA type used was “within subjects ANOVA”, significance threshold was defined as the corrected *p*-value < 0.05, and False Discovery Rate < 0.05 was chosen for multiple testing correction. ASCA was performed with the default parameters supplied by the website. HCA was employed to highlight the variation trend of differential metabolites between comparisons using the Euclidean correlation method and Ward clustering algorithm. Metabolites were selected based on these criteria: *t-*test (*p* adjusted < 0.05) and PLS-DA (VIP component 1 > 1). Pathway analysis was performed to discover the most relative pathways involving the ethanol stress response of recombinant strains respect to that of the parental. The global test algorithm and relative-betweenness centrality algorithm were specified for pathway enrichment analysis and pathway topology analysis, respectively, in pathway analysis.

## 4. Conclusions

The results of this study indicated that, once exposed to short-term ethanol stress, the engineered strains F2 and F6, previously developed by the multiple δ-integration of a glucoamylase in the industrial *S. cerevisiae* 27P, did not display any statistically significant differences in terms of mortality, with respect to their parental. Conversely, yeast differentially shaped their metabolomes, showing strain-specific patterns. FTIR resulted in a useful predictor of LC/MS analysis which indicated a huge alteration in their metabolomes towards the different pathways involved in ethanol and oxidative stress response. These findings suggest that the multiple δ-integrations prompted massive metabolomic changes in response to short-term ethanol stress. Deeper transcriptomic, metabolomic and genomic insights are therefore needed to understand how, and to what extent, genetic engineering, which can be successful in terms of the target phenotype, could strongly affect the yeast metabolome.

## Figures and Tables

**Table 1:** Mortality (%) induced by increasing ethanol concentrations on 27P, F2 and F6 strains.

	Mortality (%)		
Ethanol% (v/v)	P27	F2	F6
0	0	0	0
7.5	0	0	0
15	0	0	0
20	0	0	0
21	13	22	18
22	33	36	34
23	49	54	50
24	78	82	72
25	**100**	**100**	**100**

## Supplementary Materials

The following are available online at https://www.mdpi.com/2218-1989/10/4/140/s1, Figure S1: Major patterns associated with Phenotype and with the Interaction between Phenotype and Ethanol in the comparison between 27P *vs.* F2 strains (A) and 27P *vs*. F6 strains (B). Figure S2: Metabolites involved in the disturbance of the beta-alanine and glutathione metabolism. The bar plots on the left show the original values (mean +/- SD). The box and whisker plots on the right summarize the normalized values. Note, positive infinite numbers are represented as 999,999, and negative infinite numbers -999999. Table S1: List of metabolites identified in 27P, F2 and F6 strains under ethanol stress. Table S2: Important features identified by PLS-DA. VIP scores are calculated for each component. Table S3: Features identified by Significant features identified by advanced ANOVA in the comparison between 27P *vs.* F2 strain. Table S4: Features identified by Significant features identified by advanced ANOVA in the comparison between 27P *vs.* F6 strain. Table S5: Important features identified by ASCA in the comparison between 27P *vs.* F2 recombinant. Table S6: Important features identified by ASCA in the comparison between 27P *vs.* F6 recombinant. Table S7: Important features selected by *t*-test. Comparison between 27P *vs.* F2 recombinant. Table S8: Important features selected by *t*-test. Comparison between 27P *vs.* F6 recombinant. Table S9: Detailed results from Pathway Analysis. Comparison between 27P *vs.* F2 recombinant. The statistical *p* values from enrichment analysis are further adjusted for multiple testings. The Total is the total number of compounds in the pathway; the Hits is the actual matched number from the user-uploaded data; the Raw *p* is the original *p* value calculated from the enrichment analysis; the Holm *p* is the *p* value adjusted by Holm-Bonferroni method; the FDR *p* is the *p* value adjusted using False Discovery Rate; the Impact is the pathway impact value calculated from pathway topology analysis. Table S10: Detailed results from Pathway Analysis. Comparison between 27P *vs.* F6 recombinant. The statistical *p* values from enrichment analysis are further adjusted for multiple testings. The Total is the total number of compounds in the pathway; the Hits is the actual matched number from the user-uploaded data; the Raw *p* is the original *p* value calculated from the enrichment analysis; the Holm *p* is the *p* value adjusted by Holm-Bonferroni method; the FDR *p* is the *p* value adjusted using False Discovery Rate; the Impact is the pathway impact value calculated from pathway topology analysis.
