# Supplementary figures and images for: Delta-Integration of Single Gene Shapes the Whole Metabolomic Short-Term Response to Ethanol of Recombinant Saccharomyces cerevisiae Strains
Source: Metabolites. 2020 Apr 3;10(4):140. doi: 10.3390/metabo10040140 (PMC7241245; doi:10.3390/metabo10040140)

**Supplementary Figures**

**Figure S1.**


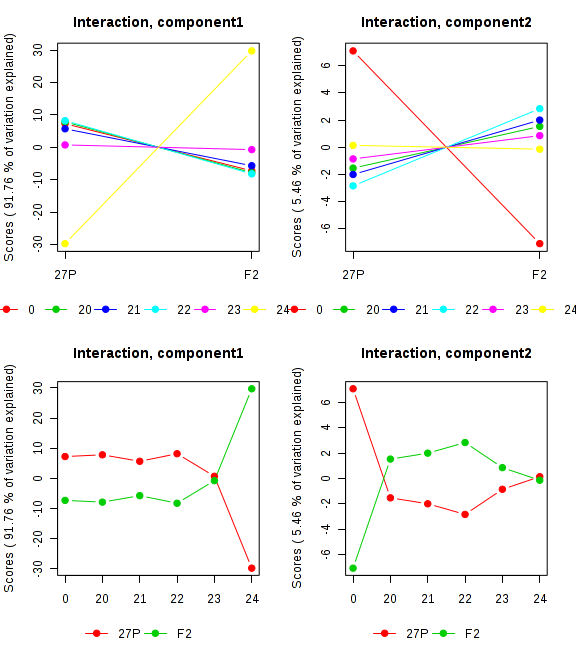

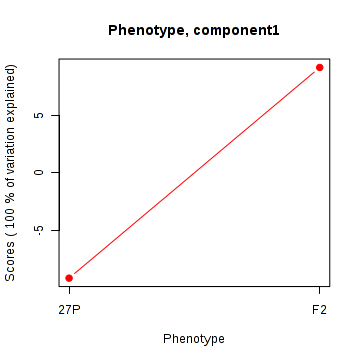

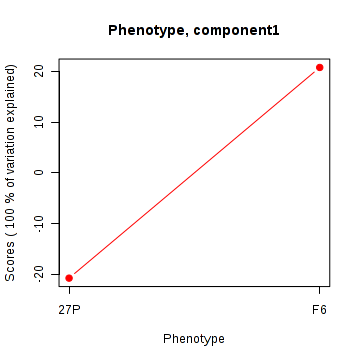

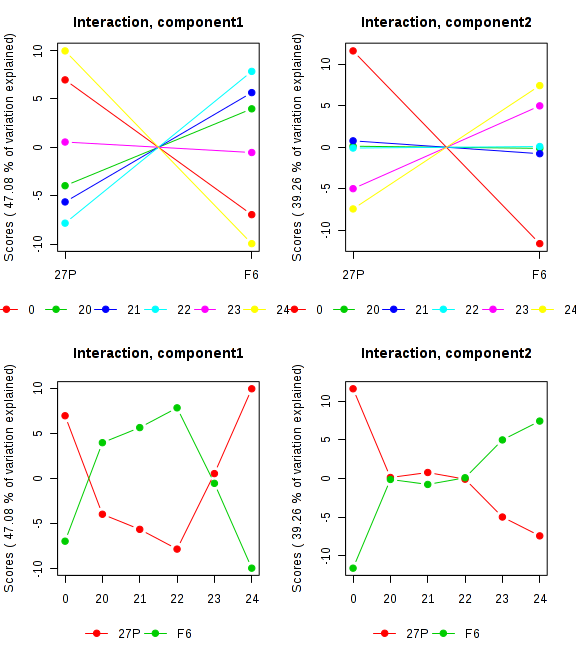


**B**

**A**

**Figure S2.**


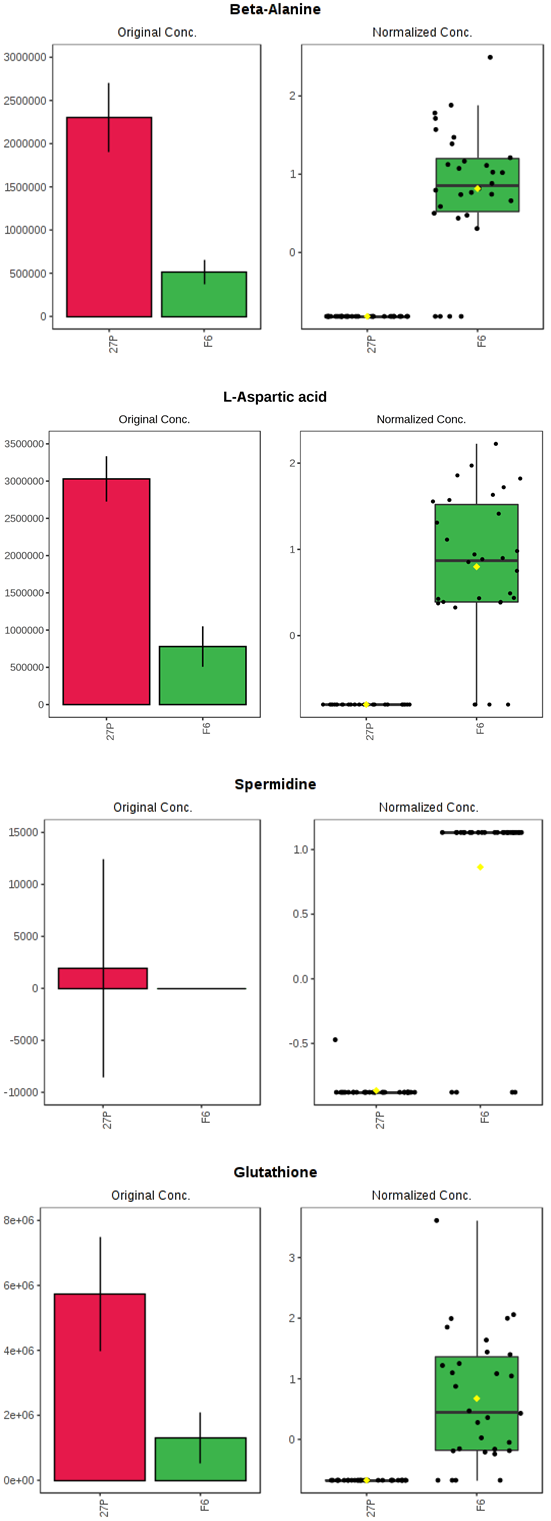

Supplement: Supplementary file 1 [file metabolites-10-00140-s001.zip › Supplementary Tables and Figures/Supplementary Figures.docx]
